# Minimalistic Cellulosome of the Butanologenic Bacterium Clostridium saccharoperbutylacetonicum

**DOI:** 10.1128/mBio.00443-20

**Published:** 2020-03-31

**Authors:** Bosmat Levi Hevroni, Sarah Moraïs, Yonit Ben-David, Ely Morag, Edward A. Bayer

**Affiliations:** a Department of Biomolecular Sciences, The Weizmann Institute of Science, Rehovot, Israel; b Department of Life Sciences, Ben-Gurion University of the Negev, Beer-Sheva, Israel; Corporación CorpoGen

**Keywords:** glycoside hydrolases, enzymatic profiling, scaffoldin, cohesin-dockerin specificity, CBM, cohesin specificity

## Abstract

Clostridium saccharoperbutylacetonicum is a mesophilic, anaerobic, butanol-producing bacterium, originally isolated from soil. It was recently reported that C. saccharoperbutylacetonicum possesses multiple cellulosomal elements and would potentially form the smallest cellulosome known in nature. Its genome contains only eight dockerin-bearing enzymes, and its unique scaffoldin bears two cohesins (Cohs), three X2 modules, and two carbohydrate-binding modules (CBMs). In this study, all of the cellulosome-related modules were cloned, expressed, and purified. The recombinant cohesins, dockerins, and CBMs were tested for binding activity using enzyme-linked immunosorbent assay (ELISA)-based techniques. All the enzymes were tested for their comparative enzymatic activity on seven different cellulosic and hemicellulosic substrates, thus revealing four cellulases, a xylanase, a mannanase, a xyloglucanase, and a lichenase. All dockerin-containing enzymes interacted similarly with the second cohesin (Coh2) module, whereas Coh1 was more restricted in its interaction pattern. In addition, the polysaccharide-binding properties of the CBMs within the scaffoldin were examined by two complementary assays, affinity electrophoresis and affinity pulldown. The scaffoldin of *C. saccharoperbutylacetonicum* exhibited high affinity for cellulosic and hemicellulosic substrates, specifically to microcrystalline cellulose and xyloglucan. Evidence that supports substrate-dependent *in vivo* secretion of cellulosomes is presented. The results of our analyses contribute to a better understanding of simple cellulosome systems by identifying the key players in this minimalistic system and the binding pattern of its cohesin-dockerin interaction. The knowledge gained by our study will assist further exploration of similar minimalistic cellulosomes and will contribute to the significance of specific sets of defined cellulosomal enzymes in the degradation of cellulosic biomass.

## Abstract (precis)

Cellulosome-producing bacteria are considered among the most important bacteria in both mesophilic and thermophilic environments, owing to their capacity to deconstruct recalcitrant plant-derived polysaccharides (and notably cellulose) into soluble saccharides for subsequent processing. In many ecosystems, the cellulosome-producing bacteria are particularly effective “first responders.” The massive amounts of sugars produced are potentially amenable in industrial settings to further fermentation by appropriate microbes to biofuels, notably ethanol and butanol. Among the solvent-producing bacteria, Clostridium saccharoperbutylacetonicum has the smallest cellulosome system known thus far. The importance of investigating the building blocks of such a small, multifunctional nanomachine is crucial to understanding the fundamental activities of this efficient enzymatic complex.

## INTRODUCTION

Cellulosomes are high-molecular-weight, multienzyme complexes, which are deemed important for efficient bacterial degradation of recalcitrant polysaccharides, notably cellulose. These highly efficient complexes are produced by specific cellulolytic anaerobic bacteria (1). These specialized bacteria can be found in various environments in nature, such as soil, compost piles, sewage sludge, and the digestive tracts of insects and herbivorous animals (2). The cellulosome system is based on several unique and interacting functional and structural subunits. The functional catalytic components (i.e., the enzymatic system) are attached to noncatalytic, structural scaffoldin proteins. The assembly between the scaffoldin and enzymes occurs via the complementary interaction of noncatalytic modules called cohesins (located on the scaffoldin) and dockerins (modular component of the enzymes). Frequently, scaffoldin subunits can also contain an additional functional component, a carbohydrate-binding module (CBM). The CBM assists the bacterium in attaching to cellulose, so that the cellulosome gains proximity to both its substrate and the bacterial cell wall (1).

Cellulosome architecture can vary from “complex” cellulosomes, composed of multiple interacting scaffoldins which assemble into a much more intricate cellulosome complex (1, 2), to “simple” cellulosomes that contain a single major enzyme-bearing scaffoldin subunit. Complex cellulosome systems can be found both in thermophilic bacteria, e.g., Clostridium clariflavum, and in the mesophilic *Bacteroides* (*Pseudobacteroides*) *cellulosolvens*. The C. clariflavum cellulosomal system has 13 scaffoldins and 75 dockerin-containing proteins (mainly enzymes) (3), whereas B. cellulosolvens has 212 dockerin-bearing proteins and 32 scaffoldins (4). On the other hand, much simpler and smaller cellulosome systems can be found in the mesophilic bacteria Clostridium bornimense and Clostridium acetobutylicum, which have 5 dockerins and 2 scaffoldins in the former and 10 dockerins and 2 scaffoldins in the latter (5). In addition to the major scaffoldin, which bears multiple cohesins and one or two CBMs, the latter mesophilic cellulosome-producing bacteria each contains a gene termed *orfX* encoding a single cohesin. This type of protein can thus also be considered a scaffoldin, but its precise role in cellulosome assembly is unclear (6).

In recent years, genome sequencing of microorganisms has become more accessible and standardized. This progress contributes to the discovery of new cellulosomal species, thus expanding our knowledge of the cellulosomal paradigm in nature. In a recent review, a genome-wide analysis of a dozen mesophilic clostridial species revealed the presence of heretofore undescribed cellulosome systems in three mesophilic clostridia (5). In particular, the cellulosomal system of Clostridium saccharoperbutylacetonicum was of special interest to us. C. saccharoperbutylacetonicum is a mesophilic, anaerobic, spore-forming, butanol-producing Gram-positive bacterium originally isolated from soil (7), and its potential to use lignocellulosic substrates was never reported. There are several other known solvent-producing clostridia, such as C. acetobutylicum, C. saccharobutylicum, C. beijerinckii, etc. Among these solventogenetic clostridia, only C. acetobutylicum and *C. saccharoperbutylacetonicum* are known to possess cellulosomal elements. However, despite the presence of major dockerin-bearing cellulases, the cellulosome of C. acetobutylicum was reported to be inactive on cellulosic substrates, and the extracellular cellulolytic activity was limited and/or regulated by the available substrate (8–10). Surprisingly, the cellulosome system of *C. saccharoperbutylacetonicum* was never properly examined. Thus, we were intrigued by this bacterium and its cellulosomal system, which is so small and simple and might shed light on the basic elements of a cellulosome.

**FIG 1:**
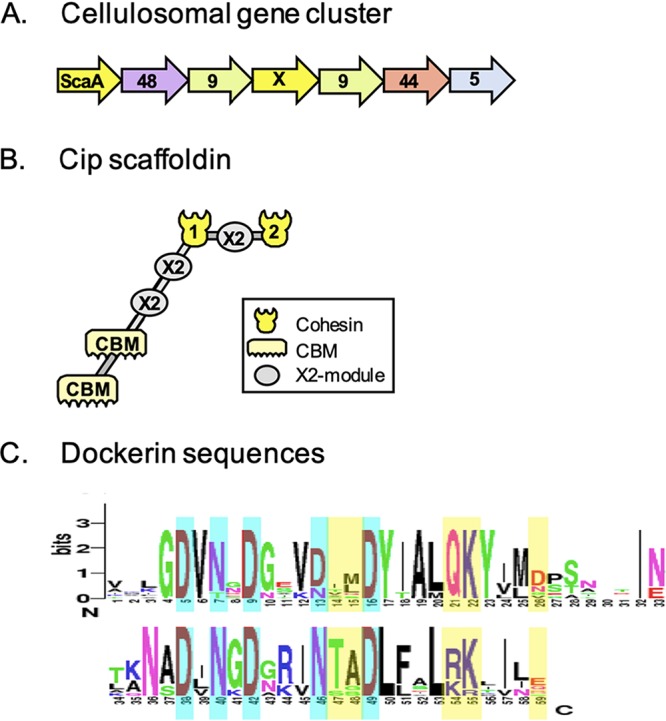
The cellulosome system of *C. saccharoperbutylacetonicum* (5). (A) Schematic representation of the cellulosome gene cluster. The gene encoding the major scaffoldin is followed downstream by genes coding for dockerin-containing cellulolytic enzymes. The major scaffoldin gene is designated *scaA*; the numbers denote the GH family; X stands for the *orfX* gene (a similar gene encoding a cohesin-containing protein is present in all cellulosome-producing bacteria). (B) Schematic representation of the modular composition of the primary scaffoldin protein. (C) Conserved sequence features of dockerin modules, visualized by WebLogo. Ca^2+^-binding residues are shown on cyan background, and putative cohesin recognition positions are shown on yellow background. Cohesin recognition residues (XXQK/TARK) are not repeated in symmetric fashion, thus suggesting a single rather than a dual binding mode of interaction. The recognition residues are unique in the current collection of dockerin sequences.

The complete and fully annotated genome of *C. saccharoperbutylacetonicum* strain N1-4 (HMT) was published in 1999, and it was found to comprise two replicons, a chromosome, and a circular megaplasmid (11). The solventogenic *sol* operon of *C. saccharoperbutylacetonicum* is located on the chromosome, as in the noncellulosome producer, Clostridium beijerinckii, but in contrast to that of Clostridium acetobutylicum, which is located on the megaplasmid. Like C. beijerinckii, *C. saccharoperbutylacetonicum* possesses an aldehyde dehydrogenase (*ald*) gene, whereas C. acetobutylicum has an alcohol/aldehyde dehydrogenase-encoding gene (*adhE*) together with acetoacetate decarboxylase (*adc*), which are located on separate operons (12). The recently reported cellulosomal elements of *C. saccharoperbutylacetonicum* (5) are located in the cellulosome gene cluster of the chromosome. Theoretically, its cellulosome is among the smallest known in nature, comprising a “minimalistic” cellulosome with only eight dockerin-bearing enzymes (five of which are located in the major cellulosome gene cluster (Fig. 1A), and the remaining three are distributed elsewhere on the chromosome) and only two cohesins in its single major scaffoldin (Fig. 1B). To date, this represents the smallest number of cellulosomal scaffoldin-borne cohesins among the mesophilic clostridia (5). For comparison, C. acetobutylicum which is considered to have a small and simple cellulosome, has 10 dockerin-containing enzymes and 5 cohesins on its major scaffoldin. Interestingly, *C. saccharoperbutylacetonicum* major scaffoldin contains two copies of CBM3 at the N terminus—similar to the major scaffoldin in *C. bornimense* but unlike all other known mesophilic cellulosome-producing bacteria which have only a single scaffoldin-borne CBM3. The *C. saccharoperbutylacetonicum* scaffoldin also bears three X2 domains (a module of ca. 100 amino acid residues of unknown structure and function) (13) between its CBM3 and cohesin modules (similar only to the major scaffoldin of C. acetobutylicum) (5). In addition, the *C. saccharoperbutylacetonicum* genome encodes 146 free glycoside hydrolase (GH) enzymes.

In this study, we aimed to reveal the full architecture and function of *C. saccharoperbutylacetonicum* cellulosome system. Therefore, each cellulosome-related module (cohesin, dockerin, catalytic, and carbohydrate-binding modules) was cloned, expressed in Escherichia coli, and purified, and the recombinant components were tested for enzymatic activity on cellulosic and hemicellulosic substrates. The binding capacity of each cohesin and dockerin module was tested using enzyme-linked immunosorbent assay (ELISA)-based techniques in order to evaluate their pattern of interaction. In addition, the CBMs of the scaffoldin and of the cellulosomal enzymes were explored for their binding affinities.

## RESULTS

### The cellulosomal system of *C*. *saccharoperbutylacetonicum*.

The bioinformatic analysis of *C. saccharoperbutylacetonicum* revealed a set of only eight putative dockerin-containing glycoside hydrolases (GHs) (Table 1), and one major scaffoldin, ScaA, which possess two family 3 CBMs, three X2 modules, and two cohesins. The eight putative enzymes belong to GH families 5, 9, 26, 44, 48, and 74. All putative dockerin-containing enzymes and the scaffoldin protein have an N-terminal signal peptide sequence, suggesting that these proteins are secreted.

**TABLE 1:** Putative dockerin-containing glycoside hydrolases of *C. saccharoperbutylacetonicum*^*a*^

GH family	Current name	Modular organization	Mol wt^*b*^	NCBI:protein accession no.
GH5	GH5A	GH5A-Doc	54,258	WP_015391959
	GH5B	GH5B-Doc	50,917	WP_015391954
GH9	GH9A	CBM4-Ig-GH9A-Doc	93,651	WP_015391950
	GH9B	GH9B-CBM3c-Doc	77,973	WP_015391952
GH26	GH26A	CBM6-GH26A-Doc	40,989	WP_015391960
GH44	GH44A	GH44A-Doc	65,499	WP_015391953
GH48	GH48A	GH48A-Doc	81,337	WP_015391949
GH74	GH74A	GH74A-Doc	90,254	WP_015391947

a Abbreviations: GH, glycoside hydrolase; Doc, dockerin; CBM, carbohydrate-binding module; Ig, immunoglobulin-like domain of unknown function.

b The molecular weight based on the known amino acid composition of the desired protein using the ProtParam tool (15).

In order to characterize the *C. saccharoperbutylacetonicum* cellulosomal components, the following proteins were cloned (without their signal peptide sequence) and expressed: the eight dockerin-containing enzymes GH5A, GH5B, GH9A, GH9B, GH26A, GH44A, GH48A, and GH74A and the putative scaffoldin (ScaA). Since the X2 modules could have a stabilizing role for the neighboring cohesin (14), the two ScaA cohesins were also cloned and expressed separately with and without their X modules as follows: Coh1, X2-Coh1, Coh2, and X2-Coh2. All proteins were purified to homogeneity with a major band on sodium dodecyl sulfate-polyacrylamide gel (SDS-PAG) at the expected calculated molecular weights (see Fig. S1 in the supplemental material).

10.1128/mBio.00443-20.1 FIG S1 Purity of the recombinant enzymes after purification as assessed by SDS-PAGE (10% acrylamide gels). The molecular weights (in kilodaltons) for the enzymes are as follows: scaffoldin, 123; Coh1, 35; X-Coh1, 35; Coh2, 34; X-Coh2, 34; GH5A, 63; GH5B, 45; GH9A, 54; GH9B, 67; GH26A, 44; GH44A, 34; GH48A, 44; GH74A, 44. Download FIG S1, JPG file, 0.2 MB. Copyright © 2020 Levi Hevroni et al. 2020 Levi Hevroni et al. This content is distributed under the terms of the Creative Commons Attribution 4.0 International license.

### Activity profiling of *C. saccharoperbutylacetonicum* cellulosomal enzymes.

In order to identify the preferred substrate(s) of the *C. saccharoperbutylacetonicum* dockerin-containing enzymes, all enzymes were tested for their activity on various cellulosic and hemicellulosic substrates, according to their putative activity (as reported for their respective GH family [15]). This approach was used previously to gain initial assessment of enzyme activity in Ruminococcus champanellensis (16). Since the activity of an enzyme is influenced by environmental conditions (e.g., pH, temperature) (17), an optimization process was first performed to determine optimal conditions of activity.

### Optimizing the activity assay parameters.

For the optimization process of the enzymatic activity assays, we chose two representative enzymes, GH44A (originally considered a putative cellulase) and GH5B (a putative mannanase). Each enzyme was tested with its corresponding substrate: GH44A was tested for its ability to cleave carboxymethyl cellulose (CMC) and GH5B to cleave locust bean gum (mannan). The enzymes were active on each substrate in accordance with the other characterized members of the same GH families (15). In order to estimate the optimal parameters for enzyme activity (e.g., pH, temperature), four temperatures were tested: 30, 40, 50, and 60°C. We also tested three buffers at different pH values (according to the buffer type): acetate buffer pH 4 and 5, citrate buffer pH 5 and 6, and phosphate buffer pH 6 and 7. Our results revealed that the highest activity obtained for both enzymes was with the acetate buffer under conditions of pH 5 at 40°C; therefore, these conditions were selected for the biochemical characterization of the other enzymes. The results for GH5B and GH44A are presented in Fig. S2A and S2B, respectively.

10.1128/mBio.00443-20.2 FIG S2 Enzymatic activity of GH5B (A) and GH44A (B) under different buffer/temperature/pH conditions for optimization of the parameters. The comparative enzymatic activities were carried out at a concentration of 0.5 μM, an agitation speed of 300 rpm for 2 h with 2% CMC for GH5B and 2% mannan for GH44A. Download FIG S2, JPG file, 0.3 MB. Copyright © 2020 Levi Hevroni et al. 2020 Levi Hevroni et al. This content is distributed under the terms of the Creative Commons Attribution 4.0 International license.

### Enzymatic activity profile.

**FIG 2:**
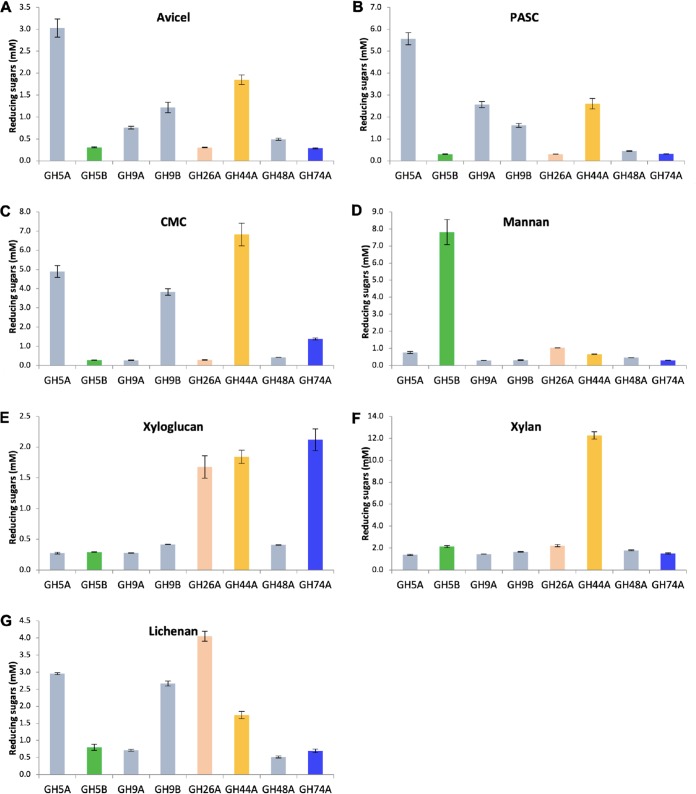
Enzymatic profiling of *C. saccharoperbutylacetonicum* cellulosomal glycoside hydrolases. The comparative enzymatic activities were carried out at a concentration of 0.5 μM in acetate buffer (pH 5) at 40°C. (A) Cellulase activity on 5% Avicel for 24 h. (B) Cellulase activity on PASC for 24 h. (C) Cellulase activity on 1% CMC for 2 h. (D) Mannanase activity on 1% locust bean gum for 2 h. (E) Xyloglucanase activity on 0.5% xyloglucan for 1 h. (F) Xylanase activity on1% beech wood xylan for 2 h. (G) Lichenase activity on 0.25% lichenan for 1 h. Reactions were performed at least twice in triplicate; standard deviations are indicated by the error bars.

The substrates tested were in accordance with the putative activity of the enzymes according to the CAZy classification (15), and all of the purified recombinant enzymes were tested for their activity(ies) on all of the tested substrates. The enzymes GH5A, GH9A, GH9B, GH44A, and GH48A were all predicted to be cellulases according to CAZy and bioinformatic analysis. Therefore, they were expected to show activity on some or all of the tested cellulosic substrates: Avicel (microcrystalline cellulose), phosphoric acid-swollen cellulose (PASC), and CMC. Indeed, all of the putative cellulases exhibited activity on Avicel and PASC (Fig. 2A and B). GH5A gave the highest activity on both substrates. GH44A also presented high activity, whereas GH9A and GH9B showed moderate activity. GH48A showed very low activity on all cellulosic substrates, as expected for this particular putative exoglucanase, based on previous performance of the GH48s from other bacterial species (18–21). GH9B exhibited higher relative activity on Avicel versus PASC. Among the five predicted cellulases, three were highly active on the soluble CMC substrate: GH5A, GH9B, and GH44A (Fig. 2C). Therefore, these three can be classified as endoglucanases or processive endoglucanases (the GH9B enzyme). In contrast, GH9A and GH48A showed only nominal activity on CMC versus Avicel and PASC and are thus considered exoglucanases.

The two putative xyloglucanases, GH44A and GH74A, were both active on xyloglucan, but GH74A was more active than GH44A (Fig. 2E). GH44A was also very active on all cellulosic substrates tested and exhibited the highest activity on CMC of all of the *C. saccharoperbutylacetonicum* enzymes. Nevertheless, GH44A was the only enzyme that was highly active on beech wood xylan (Fig. 2F) and was therefore classified as a xylanase, while GH74A was classified as a xyloglucanase. Intriguingly, GH44A exhibits a very broad activity pattern with the ability to cleave all of the tested hemicellulosic and cellulosic substrates tested, with the exception of mannan (Fig. 2). The observed xylanase activity in the *C. saccharoperbutylacetonicum* GH44 enzyme reinforces previous reports (22, 23), thus extending the CAZy annotation (15) and previously reported work (24) that indicate endoglucanase and xyloglucanase activities for this family of enzymes.

Surprisingly, of the two predicted mannanases (according to their sequence and GH family affiliation), GH5B and GH26A, only GH5B was able to degrade locust bean gum mannan and showed extremely high activity on this substrate (Fig. 2D). The other putative mannanase, GH26A, was not active on the mannan substrate; however, it exhibited substantial xyloglucanase activity (Fig. 2E). We thus explored its amino acid sequence more thoroughly and constructed a phylogenetic tree (Fig. S3) to examine its genetic relationship to various characterized GH26s. The vast majority of family GH26 enzymes are indeed annotated as mannanases and the rest are annotated as xylanases. Interestingly, we discovered that GH26A has the closest sequence similarity to Lic26A from *C. thermocellum*, the single reported lichenase from family GH26 (25). Therefore, we tested the ability of all our enzymes to cleave lichenan (Fig. 2G), and indeed, GH26A presented the highest activity among all the enzymes on this substrate and could thus be classified as a lichenase but it exhibited high xyloglucanase activity as well. Endoglucanases GH5A, GH9B, and GH44A also presented substantial activity on lichenan.

10.1128/mBio.00443-20.3 FIG S3 Phylogenetic tree of various GH members from family 26. GH26A from *C. saccharoperbutylacetonicum* shows the closest relationship to Lic26A from *C. thermocellum*. Organism abbreviations are as follows: Celja, Cellvibrio japonicus; Bacov, Bacteroides ovatus; Bacsu, Bacillus subtilis; Bacli, Bacillus licheniformis; Breve, Brevundimonas vesicularis; Vibsp, *Vibrio* sp.; Psesp, *Pseudomonas* sp.; Alcsp, *Alcanivorax* sp., Rumch, Ruminococcus champanellensis; Cloth, *Clostridium thermochellum*; Cloce, Clostridium cellulolyticum; Fibsu, Fibrobacter succinogenes; Bacfr, *Bacillus fragilis*; Closa, Clostridium saccharoperbutylacetonicum. The enzymes are indicated in color as follows: mannanase (green), xylanase (blue), and lichenase (black). Download FIG S3, JPG file, 0.2 MB. Copyright © 2020 Levi Hevroni et al. 2020 Levi Hevroni et al. This content is distributed under the terms of the Creative Commons Attribution 4.0 International license.

**FIG 3:**
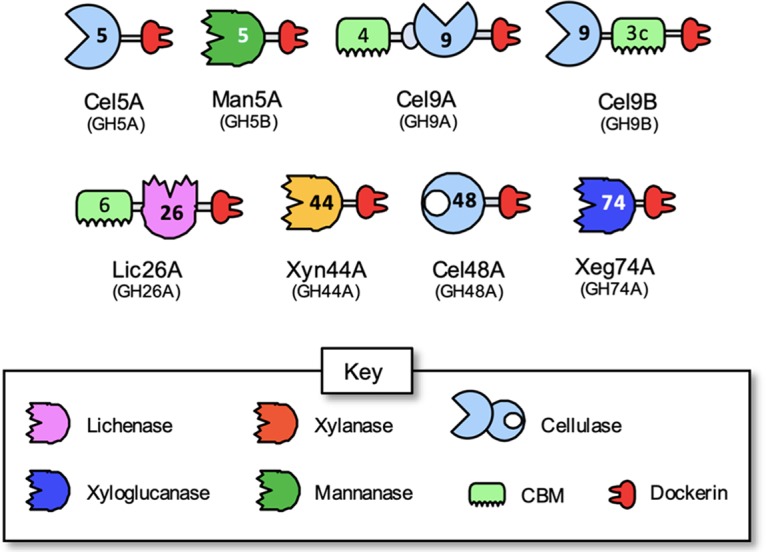
Schematic representation and proposed nomenclature of the experimentally defined dockerin-containing enzymes of *C. saccharoperbutylacetonicum*. Enzymatic activities are color coded according to the key. GH and CBM families are indicated by the numbers.

All of the cellulosomal enzymes from *C. saccharoperbutylacetonicum* were thus characterized for their activity(ies) on specific substrate(s), and four cellulases, a xylanase, a mannanase, a xyloglucanase, and a lichenase, were revealed, with the GH44 xylanase exhibiting broad specificity for several other polysaccharide substrates. In Fig. 3, the schematic modular architecture of these enzymes is shown, along with their enzymatic activities, their dockerin groupings (see paragraph below) and their proposed nomenclature.

### Cohesin-dockerin recognition.

*C. saccharoperbutylacetonicum* contains two cohesins on its scaffoldin and eight dockerin-containing enzymes that can theoretically self-assemble into cellulosomal architectures. In order to draw a complete map of the potential cohesin-dockerin interactions, all of the dockerin-containing enzymes were examined for their interactions with each of the two *C. saccharoperbutylacetonicum* cohesins (with or without their original adjoining X2 module—i.e., X2-Coh1, Coh1, X2-Coh2, and Coh2) using an ELISA-based affinity assay system (26). The two individual cohesin-containing genes were each fused to a CBM cassette, both for antibody recognition and for increased solubility of the cohesin modules when expressed in E. coli (27). The presence of the CBM could also be used for an additional purification step.

**FIG 4:**
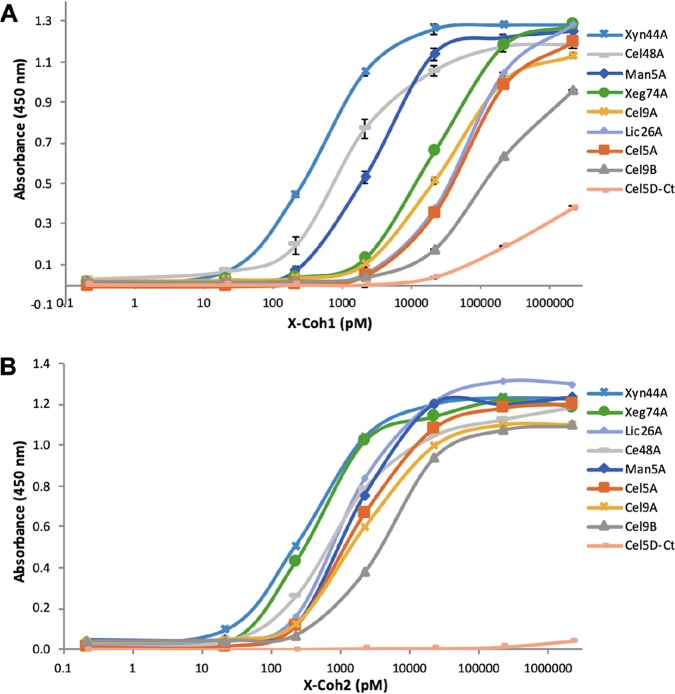
Affinity-based ELISA of cohesins of the *C. saccharoperbutylacetonicum* scaffoldin on immobilized dockerins. (A) X2-Coh1 interaction pattern. (B) X2-Coh2 interaction pattern. Cel5D-*Ct* (a dockerin-containing enzyme from *C. thermocellum*) was used as a negative control.

At first, we examined the effect of the presence of the adjacent X2 module on the binding capacities of the cohesins with their dockerin-containing enzymes. The results showed that the interactions of the dockerins were higher when the X2 module was present adjacent to both cohesins, and the effect was higher for X2-Coh1 (Fig. S4). In view of these findings, in subsequent experiments, we used cohesins fused to X2 modules for analysis. Each immobilized dockerin was tested for its binding capacity with increasing concentrations of either X2-Coh1 or X2-Coh2 (Fig. 4).

10.1128/mBio.00443-20.4 FIG S4 Affinity-based ELISA assay of Coh1 versus X-Coh1 and Coh2 versus X-Coh2 (with a concentration of 1 or 10 μg/ml) on immobilized dockerins. Download FIG S4, JPG file, 0.3 MB. Copyright © 2020 Levi Hevroni et al. 2020 Levi Hevroni et al. This content is distributed under the terms of the Creative Commons Attribution 4.0 International license.

**FIG 5:**
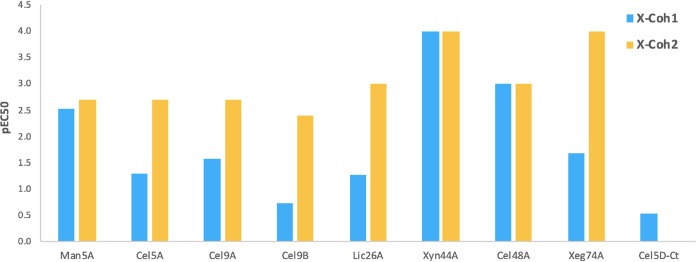
pEC_50_ values calculated for each Coh-Doc pair from their concentration-response curves presented in Fig. 4.

The results indicated different patterns of interaction for the two X2-cohesin modular dyads. We thus calculated the logarithmically transformed 50% effective concentration (pEC_50_) values for each Coh-Doc pair (Fig. 5) from the appropriate concentration-response curve presented in Fig. 4. Our data indicate that all dockerin-containing enzymes interacted strongly with the X2-Coh2 module, with Xyn44A and Xeg74A presenting the highest affinities (pEC_50_ values of 4.0) and Cel9B exhibiting the lowest affinity (pEC_50_ = 2.4). Nevertheless, it appeared that X2-Coh1 was more restricted in its interaction pattern. The dockerins from Man5A, Cel48A, and Xyn44A interacted strongly with X2-Coh1 with pEC_50_ values of 2.5, 3.0, and 4.0, respectively. The rest of the tested dockerins interacted weakly with X2-Coh1, where Cel9B exhibited the lowest binding affinity (pEC_50_ = 0.7). For a negative control, a dockerin-containing enzyme from Clostridium thermocellum (Cel5D-*Ct*) was used.

### CBM affinity.

Another interesting feature of the cellulosome is the presence of the two CBM3s on the *C. saccharoperbutylacetonicum* scaffoldin, which mediate the binding to polysaccharide substrate(s). These particular CBMs could shed light on the preferred nutritional substrate of the bacterium. *C. saccharoperbutylacetonicum* scaffoldin has two copies of the CBM3 at the N terminus of its scaffoldin gene (5). In order to experimentally evaluate the binding affinity of *C. saccharoperbutylacetonicum* scaffoldin, we used an affinity electrophoresis assay (28). We used various soluble polysaccharides (PASC, xyloglucan, xylan, starch, mannan, and lichenan) in nondenaturing polyacrylamide gels containing one of the latter polysaccharides in soluble form to test the binding abilities of a CBM. In addition to the scaffoldin CBMs, three out of the eight cellulosomal enzymes of *C. saccharoperbutylacetonicum* contain a CBM: Cel9A contains a CBM4 at its N terminus, Cel9B contains a CBM3c between the catalytic site and the dockerin, and Lic26A contains a CBM6 (Table 1 and Fig. 3).

In this binding assay, the CBM binding ability to the tested polysaccharide is evaluated by the delayed migration of the protein through the gel containing the polysaccharide compared to a comparable gel that lacks the polysaccharide. A protein lacking a CBM could also be delayed due to the inherent affinity of its catalytic site to the polysaccharide (29, 30). Relative mobilities (migration distance of the protein on the gel compared to its migration on a gel with the tested polysaccharide [28]) of each protein was determined with a control gel that does not contain any polysaccharide, and bovine serum albumin (BSA) was used as a negative nonbinding control and served to normalize the relative mobilities. Gel results are presented in Fig. S5, and relative mobility values are presented in Table 2. The results show that the mobility of the *C. saccharoperbutylacetonicum* scaffoldin is hampered when run on gels with PASC and xyloglucan, meaning its CBMs demonstrated high cellulose- and xyloglucan-binding affinity. Very low relative mobility values of the scaffoldin were calculated when run on gels with 0.1% PASC (Fig. S5B) and 0.1% xyloglucan (Fig. S5D) with 0.44 and 0.56 values, respectively. Upon increasing the percentage of xyloglucan in the gel, the relative mobility of the scaffoldin further decreased to 0.33 with 0.2% xyloglucan (Fig. S5E). Although these two CBMs are classified as CBM3s (according to sequence), and would bind primarily to crystalline cellulose according to the literature (15), it seems that one or both of these CBMs are capable of binding cellulosic substrates as well as xyloglucan. Binding of CBM3s to xyloglucan was indeed demonstrated recently (31). The data showed that the CBMs bound to both crystalline cellulose and xyloglucan, thus demonstrating a broader specificity pattern than previously recognized.

**TABLE 2:**
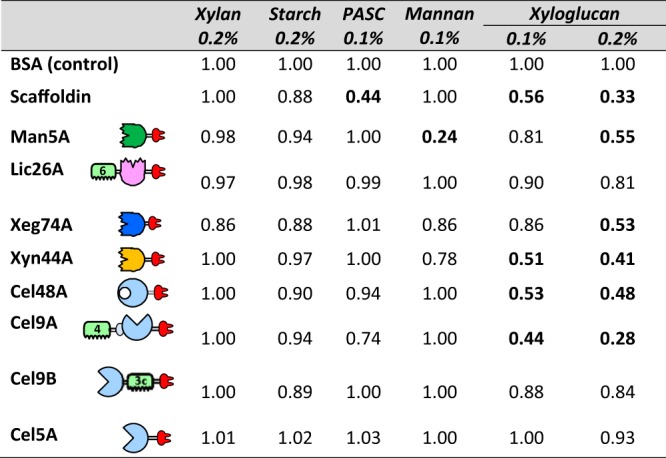
Relative mobility values of *C. saccharoperbutylacetonicum* dockerin-containing enzymes and scaffoldin (normalized to BSA) with various soluble polysaccharides

10.1128/mBio.00443-20.5 FIG S5 Affinity electrophoresis of *C. saccharoperbutylacetonicum* scaffoldin and its dockerin-containing enzymes (compared to BSA) on a control gel in the absence of a polysaccharide (A, C, F, H, and J) or in the presence of a polysaccharide. The polysaccharides were 0.1% (wt/vol) PASC (B), 0.1% (wt/vol) xyloglucan (D), 0.2% (wt/vol) xyloglucan (E), 0.2% (wt/vol) xylan (G), 0.2% (wt/vol) starch (I), 0.1% (wt/vol) mannan (K). Samples in panels A to I were loaded in the following order starting at the leftmost lane: BSA, Man5A, Lich26A, Xeg74A, Xyn44, Cel48A, Cel9A, Cel9B, Cel5A, and Sca. Samples in panels J to K were loaded in the following order from the leftmost lane: BSA, Sca, Cel5A, Cel9B, Cel9A, Cel48A, Xyn44, Xeg74A, Lich26A, and Man5A. Download FIG S5, JPG file, 0.4 MB. Copyright © 2020 Levi Hevroni et al. 2020 Levi Hevroni et al. This content is distributed under the terms of the Creative Commons Attribution 4.0 International license.

Among the enzymes, Cel5A, Cel9B (with CBM3c), and GH26A (which contains an N-terminal CBM6) did not display any significant binding affinity on any of the tested polysaccharides. Cel9A (which contains an N-terminal CBM4) showed low mobility on 0.1% PASC and even lower mobility on xyloglucan, with relative mobility values of 0.74 and 0.28 (on 0.2% xyloglucan), respectively. Some of the enzymes that do not contain a CBM also exhibited some retention on the various gels. For example, Xyn44A and Xeg74A both demonstrated low mobility on xyloglucan, with relative mobility values of 0.41 and 0.53, respectively (on 0.2% xyloglucan), and both enzymes were found to be highly active on this substrate (Fig. 2E). Man5A was also delayed on the xyloglucan gel, with a relative mobility of 0.55 (on 0.2% xyloglucan). The relative mobility of Man5A on its main substrate, mannan, was very low—0.24. Cel48A, which was classified earlier as an exoglucanase, exhibited low mobility on PASC and xyloglucan with relative mobility values of 0.35, and 0.48 (on 0.2% xyloglucan), respectively. As expected, BSA (the negative control) did not bind to any of the polysaccharides.

### Affinity pulldown assay.

To complement the affinity electrophoresis assays that shed light on the affinity of the CBMs and related modules to the soluble polysaccharides, we used an affinity pulldown assay to provide insights on binding to insoluble polysaccharides. Using this method, the tested CBM is incubated with an insoluble polysaccharide, and after a centrifugation step, the concentration of bound (pellet) and unbound protein (supernatant fluids) is measured. Here, we tested the ability of the *C. saccharoperbutylacetonicum* scaffoldin to bind the insoluble cellulosic substrate Avicel. The CBM3 of *C. thermocellum* was used as a positive control. Our results clearly demonstrated that the *C. saccharoperbutylacetonicum* scaffoldin has the ability to bind Avicel (Fig. S6), with 46% bound protein versus 50% for CBM3. These results are with agreement with the affinity electrophoresis results (Table 2).

10.1128/mBio.00443-20.6 FIG S6 Affinity pulldown results. The *C. saccharoperbutylacetonicum* scaffoldin, *C. thermocellum* CBM3a (as a positive control), and BSA (as a negative control) were incubated with Avicel. The concentration of proteins in the bound and unbound fractions were measured by BCA assay. Download FIG S6, JPG file, 0.1 MB. Copyright © 2020 Levi Hevroni et al. 2020 Levi Hevroni et al. This content is distributed under the terms of the Creative Commons Attribution 4.0 International license.

### Detection of *C. saccharoperbutylacetonicum* scaffoldin *in vivo*.

Next, we wanted to explore the ability of *C. saccharoperbutylacetonicum* to secrete a cellulosome *in vivo*. The results we described demonstrated that the *C. saccharoperbutylacetonicum* cellulosome targets mainly cellulose but also other hemicellulose substrates, specifically mannan, xylan, xyloglucan, and lichenin (Fig. 2 and 3 and Table 2). It has been reported that *C. saccharoperbutylacetonicum* and other butanologenic bacteria do not ferment purified cellulose (32, 33). Therefore, in order to explore *in vivo* cellulosome secretion by *C. saccharoperbutylacetonicum*, we chose to grow the bacterium on two different and contrasting carbohydrate sources. These two sources included a simple soluble saccharide substrate, i.e., the disaccharide cellobiose (CB) which is the major breakdown product of cellulose, and a natural complex insoluble cellulosic substrate, i.e., brewer’s spent grain (BSG) (34, 35).

**FIG 6:**
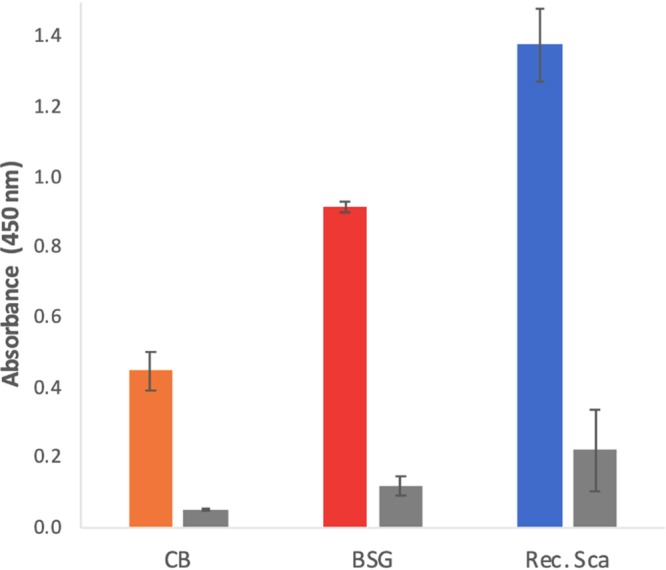
*In vivo* affinity-based ELISA of *C. saccharoperbutylacetonicum* secreted scaffoldin (cellulosome) when grown on media containing cellobiose (CB) or brewer’s spent grain (BSG). The recombinant scaffoldin (blue column) was used as a positive control. Absorbance at 450 nm represents the amount of XynDoc44, which corresponds to the relative amount of scaffoldin in the samples. Negative-control experiments (gray columns) were performed without the XynDoc44 interaction step.

In order to detect the secreted cellulosome, we applied a modification of the affinity-based ELISA approach (26) used above. Here, we used the concentrated spent cell culture media and immobilized their contents to an ELISA plate. In order to confirm the presence of the native scaffoldin in the immobilized material, we interacted the samples with a recombinant dockerin-bearing probe (XynDoc44) that exhibited the highest affinity toward both cohesins in the cohesin-dockerin recognition section (Fig. 4 and 5). The extent of interacting dockerin was determined immunochemically using an antixylanase primary antibody and horseradish peroxidase (HRP)-labeled secondary antibody as reported previously (26). Results presented in Fig. 6 show the relative amount of scaffoldin (cellulosome) in the samples, compared to a positive control (the recombinant scaffoldin ScaA). The results display a larger amount of secreted scaffoldin when the bacterium was grown on the natural cellulosic substrate compared to growth of the bacterium on the simple soluble disaccharide, thus providing experimental evidence that *C. saccharoperbutylacetonicum* actually secretes cellulosomal components *in vivo*.

In order to validate the results obtained from the affinity-based ELISA approach, we also employed an affinity pulldown assay used above, where we exploited the ability of the recombinant CBM-bearing scaffoldin to bind microcrystalline cellulose (Avicel). Using this approach, we employed a modified version of the pulldown assay, where we incubated the concentrated spent cell culture media with Avicel to “fish out” the native scaffoldin from the tested samples. The results (Fig. S7) confirm the presence of a larger amount of scaffoldin secreted when the bacterium was grown on BSG versus CB. These results correlate well with those obtained by the affinity-based ELISA (Fig. 6).

10.1128/mBio.00443-20.7 FIG S7 *In vivo* affinity-based ELISA of *C. saccharoperbutylacetonicum*-secreted scaffoldin (cellulosome) when grown on media containing cellobiose (CB) or brewer’s spent grain (BSG). The recombinant scaffoldin (blue column) was used as a positive control. Samples were selectively immobilized on microcrystalline cellulose (Avicel). Absorbance at 450 nm represents the amount of XynDoc44, which corresponds to the relative amount of scaffoldin in the samples. Download FIG S7, TIF file, 0.4 MB. Copyright © 2020 Levi Hevroni et al. 2020 Levi Hevroni et al. This content is distributed under the terms of the Creative Commons Attribution 4.0 International license.

## DISCUSSION

Current lignocellulosic biofuel research mainly focuses on bioethanol, aiming at replacing starch- and other simple-sugar-based raw materials, especially from corn, which has caused the “Food versus Fuel” controversy (36). However, ethanol has many disadvantages as a biofuel. It has a low energy density (less than two-thirds that of gasoline) (37), it is highly hygroscopic and corrosive, and thus cannot be transported and stored in existing pipelines and storage facilities. Moreover, vehicles cannot be powered by pure ethanol without redesigning the engines in order to use the low-energy fuel. Therefore, the prospect for large-scale use of ethanol to replace petroleum fuels is not promising. On the other hand, biobutanol has superior fuel properties (higher energy density, lower volatility, etc.), and can fit better into existing fuel infrastructures (with low water miscibility and corrosiveness). Biobutanol is safer (low flammability) than ethanol and can directly replace gasoline in car engines without modification. It is therefore considered an advanced biofuel, superior to bioethanol (38). In addition, butanol is also a bulk fundamental material with a variety of uses, including as a precursor in organic synthesis for producing dyes, paints, plastics, resins, and rubber, as a solvent for producing antibiotics and synthetic drugs, and as a food-grade extraction agent in the food and fragrance industries. Therefore, there is a need for biobutanol production from renewable biomass.

The solvent-producing clostridia are known for their ability to grow on a variety of polysaccharides. Although *C. saccharoperbutylacetonicum* does not ferment cellulose, it can utilize other simple and more complex sugars (7, 39), including different cellulosic biowastes as carbon sources (40–42), and use them to produce biobutanol. Another unique feature of these strains is their ability to use starch directly without any additional hydrolysis step. Under these conditions, *C. saccharoperbutylacetonicum* produced 16.9 g/liter butanol from cassava starch (43).

Since the discovery of the cellulosome, many cellulosomal systems have been investigated from “complex” systems such as the cellulosome of the extensively researched *C. thermocellum* (44) and the largest and highly complexed cellulosome of *B. cellulosolvens* (4) to much smaller and “simple” systems (5) such as those of *C. josui* (45) and the solvent-producing C. acetobutylicum (10). Here, we conducted a broad analysis of the minimal cellulosome of *C. saccharoperbutylacetonicum*, which currently comprises the smallest known cellulosome thus far.

The *C. saccharoperbutylacetonicum* cellulosome contains eight dockerin-containing enzymes that were characterized in the present study. In addition, the putative *C. saccharoperbutylacetonicum* scaffoldin was also characterized as a complete unit as well as its individual components, i.e., the cohesin modules separately (i.e., Coh1 and Coh2 with and without their adjacent X modules). Initially, according to sequence, we inferred that the eight cellulosomal enzymes would exhibit the predicted activities of two mannanases, four cellulases, and two xyloglucanases. However, after conducting broad activity profiling using model substrates, we discovered that some of the experimentally derived activities deviated from initial predictions, thus revealing five different types of enzyme activities and not three (with the addition of lichenase and xylanase activities).

*C. saccharoperbutylacetonicum* possesses a set of eight distinct enzymes with predicted cellulase (endoglucanase, processive endoglucanase, and exoglucanase) and hemicellulase activities. The hemicellulases, GH26, GH44, GH74, and the exoglucanase GH48, each appear once in the genome, and they are all cellulosomal (containing a dockerin module). The GH9 and GH5 enzymes have three and four members, respectively, in the *C. saccharoperbutylacetonicum* genome, where two of each family are cellulosome related. Among the four cellulosomal cellulases, two were characterized as a standard endoglucanase and a processive endoglucanase (Cel5A and Cel9B, respectively), whereas the other two were classified as exoglucanases (Cel9A and Cel48A). Intriguingly, Lic26A, Xyn44A, and Xeg74A were all able to digest xyloglucan, where Xeg74A exhibited the highest activity. It was also surprising to discover a lichenase among this small selection of enzymes. *C. saccharoperbutylacetonicum* Lic26A is the second known published lichenase of the GH26 family. Interestingly, Xyn44A might be an interesting candidate for designer cellulosome (46–48), in view of its high activity on xylan while also being active on a diverse set of cellulolytic and hemicellulolytic substrates. It also interacted strongly with both cohesins. Moreover, Xyn44A was active and stable at 60°C (see Fig. S2 in the supplemental material). One of the most encouraging results of this paper is the measurable activity shown by Cel48A. While it is known that Cel48 of C. acetobutylicum is inactive or has minimal activity (10), it seems that this key cellulase is indeed active in *C. saccharoperbutylacetonicum*.

Although *C. saccharoperbutylacetonicum* scaffoldin has only two cohesins, they exhibited remarkably different binding patterns. Coh1 presented varied affinities toward the dockerin-containing enzymes, with the highest affinity toward Xyn44A (pEC_50_ = 4.0) and the lowest affinity toward Cel9B (pEC_50_ = 0.7). Coh2 was much less selective and exhibited higher affinity toward all dockerins (pEC_50_ range of 2.4 to 4.0). In addition, we can conclude that the Man5A, the important broad-activity Xyn44A, and the key endoglucanase Cel48A all bind very similarly to either of the two cohesin modules, whereas the other dockerins bind preferentially to Coh2 compared to Coh1. This may reflect the likelihood that two different enzymes will pair together on the same scaffoldin. Therefore, while Man5A, Xyn44A, and Cel48A will probably bind to Coh1, the other cellulases (Cel5A, Cel9A, and Cel9B), the mannanase (Man5A) and the lichenase (Lic26A) will tend to bind to Coh2.

As mentioned above, the *C. saccharoperbutylacetonicum* dockerin modules exhibit a unique feature. While the majority of the known type I dockerins interact with their corresponding cohesins via a dual binding mode (49), the *C. saccharoperbutylacetonicum* dockerins apparently display a single mode of binding (50), owing to their unique asymmetric sequences (Fig. 1C). The functional significance of this single binding mode to the *C. saccharoperbutylacetonicum* cellulosome is not currently known.

Another unusual feature of *C. saccharoperbutylacetonicum* is the presence of two copies of CBMs on its scaffoldin. Among the recently evaluated mesophilic cellulolytic clostridia (5), *C. saccharoperbutylacetonicum* and *C. bornimense* are the only known bacteria that exhibit two copies (rather than one) of a CBM3 at the N terminus of their scaffoldin gene. Interestingly, the cohesin sequences of the two latter bacterial strains are also very similar (5). Multiple CBMs may reflect a necessity to bind to longer portions of the polysaccharide chain. Affinity electrophoresis revealed that the *C. saccharoperbutylacetonicum* scaffoldin can bind both Avicel (cellulose) and xyloglucan (hemicellulose), which further supports a recently published report (31) that demonstrated that the *C. thermocellum* CBM3a does not exclusively bind to crystalline cellulose as previously thought but that it also binds to xyloglucan. In the latter report, the authors showed that the same aromatic residues of the polysaccharide recognition site are responsible for binding both crystalline cellulose and xyloglucan.

In addition to the 8 cellulosomal dockerin-bearing GHs discussed in this paper, *C. saccharoperbutylacetonicum* encodes an additional 138 free GHs (5). Among them are two conspicuous families, GH1 (putative β-glycosidases) with 20 members and GH13 (putative amylases or pullulanases) with 14 members. Since synergy between free and cellulosomal paradigms within the same microorganism has been demonstrated (51, 52), we assume that the minimalistic *C. saccharoperbutylacetonicum* cellulosome plays a supportive role to the much larger collection of free enzymes in this bacterium in deconstructing fiber biomass.

In this work, we also provide initial evidence that *C. saccharoperbutylacetonicum* secretes a cellulosome *in vivo*. Since the bacterium does not ferment cellulose, it was grown on two different types of substrates in the growth media, which were chosen according to the cellulosome function *in vitro*, as described above, in order to examine its influence on cellulosome secretion. The results suggest that the secretion is substrate dependent, whereby larger amounts of cellulosome were obtained when the bacterium was grown on the natural cellulosic substrate versus cellobiose. This phenomenon correlates with our previously published work, which demonstrated that cellulosome production in other bacterial species was regulated by the type of substrate in the growth medium (3, 44, 53).

Our analyses described in the present communication contribute to a better understanding of simple cellulosome systems with regard to the “key players” in this reduced system and the binding pattern of its cohesin-dockerin interaction. The list of eight cellulosomal cellulase and hemicellulase enzymes in *C. saccharoperbutylacetonicum* provides a basis for the minimalistic cellulosome system that would act on cellulosic biomass. A divalent scaffoldin would by definition provide a minimal requirement for enzyme proximity and targeting effects (54), which can be further examined by assembly of even smaller artificial systems, such as designer cellulosomes (46–48). In future work, it will be important to determine the significance of such a minimalistic cellulosome to this bacterium, to its growth on complex polysaccharide biomass, and to its production of butanol as a biofuel. In other words, why is it there, what is it doing, and why is it so small?

## MATERIALS AND METHODS

### Cloning of dockerins and CBM-fused cohesins.

All related cellulosomal components, dockerin-containing enzymes, cohesins, and scaffoldin genes, were cloned from *C. saccharoperbutylacetonicum* N1-4 (HMT) genomic DNA (DSMZ 14923) using appropriate primers (Sigma-Aldrich, Israel) (see Table S1 in the supplemental material). They were all cloned without their signal peptide, which was predicted by the SignalP 3.0 server (55). GH5A, GH26A, GH44A, GH48A, GH74A, and the putative scaffoldin gene were amplified by PCR using Phusion DNA polymerase (Thermo Scientific, USA). The scaffoldin modules Coh1, X-Coh1, Coh2, X-Coh2, as well as the XynDoc44 were amplified using KAPA HiFi HotStart ReadyMix PCR kit (KAPA Biosystems, USA). PCR products were then cleaved using Fastdigest enzymes (Thermo Scientific, USA): dockerin genes were cut with NcoI and XhoI, while cohesin genes were cleaved with BamHI and XhoI restriction enzymes, respectively. Next, these PCR products were purified using the HiYield gel/PCR DNA fragments extraction kit (Real Genomics, RBC Bioscience, Taiwan). The purified restricted PCR products were then ligated to a suitable plasmid, i.e., dockerin-containing genes were ligated into pET28a (Novagen, Madison, WI, USA), and cohesin modules were ligated into CBM-fused pET28a cassette (26), using T4 DNA ligase (New England BioLabs, USA). The XynDoc gene cassette consists of xylanase T6 from Geobacillus stearothermophilus with an N-terminal His tag cloned into plasmid pET9d (Novagen Inc., Madison, WI), into which a dockerin-encoding sequence was introduced between the KpnI and BamHI restriction sites of the plasmid (26). GH5B and GH9A had the restriction sites mentioned above in their desired sequence; therefore, they were cloned using a restriction free method (56). For GH5B, first, its gene was amplified using KAPA HiFi kit in order to form a mega primer. Second, it was ligated to a closed pET28a using T4 DNA ligase to create a megaplasmid. The remaining unreacted methylated plasmids were digested by DpnI (New England BioLabs, USA). The GH9A gene was cloned using the NEBuilder HiFi DNA assembly cloning Kit (New England BioLabs, USA). The constructs were designed to contain a His tag for subsequent purification. The sequences of all plasmids were verified by DNA sequencing using the appropriate primers (Table S1) or by using pETrev and T7 primers. For longer genes, internal primers were also designed (Table S1) in order to examine the full desired cloned sequences. All plasmids were transformed to competent Escherichia coli DH5α. Polyclonal antibody against xylanase-T6 was prepared as described earlier (27). Secondary antibody-enzyme conjugate (horseradish peroxidase [HRP]-labeled goat anti-rabbit IgG) was a product of Jackson ImmunoResearch Laboratories Inc. (West Grove, PA).

10.1128/mBio.00443-20.8 TABLE S1 Primers used in this study (restriction sites represented in uppercase font). Download Table S1, PDF file, 0.1 MB. Copyright © 2020 Levi Hevroni et al. 2020 Levi Hevroni et al. This content is distributed under the terms of the Creative Commons Attribution 4.0 International license.

### Protein expression and purification.

For protein expression, the plasmids containing GH5B, GH9A, GH9B, GH74A, XynDoc44, the cohesin modules, and the putative scaffoldin, were all transformed into E. coli BL21(DE3) cells. The rest of the plasmids were transformed into BL21 Star (DE) cells. All the cells, besides GH48A, were grown in 1 liter of Luria-Bertani (LB) medium (GH48A was grown in 2 liters of LB), at 37°C to an *A*_600_ of ≈0.8 to 1. For dockerin-containing enzymes, 2 mM CaCl_2_ was added to ensure proper folding of the dockerins. Subsequently, 0.1 mM (final concentration) isopropyl-β-d-thiogalactopyranoside (IPTG) was added to induce protein expression. After overnight growth at 16°C, the cells were harvested by centrifugation at 6,000 rpm for 15 min at 10°C.

The cells were resuspended in 30 ml Tris-buffered saline (TBS) (137 mM NaCl, 2.7 mM KCl, 25 mM Tris-HCl [pH 7.4]) with 5 mM imidazole and protease inhibitor cocktail and incubated on ice. The cells were then sonicated at 45% amplitude, with 50-s pulses for 3.5 min. The cells were then centrifuged (16,000 rpm, 320 min, 10°C), and the supernatant was further purified. The CBM-cohesin-containing protein was added to 2 g of preswollen macroporous beaded cellulose (Iontosorb, Czech Republic) and incubated while rotating for 1 h at 4°C. The mixture was then loaded onto a gravity column, washed with 100 ml of TBS containing 1 M NaCl, and then washed with 100 ml of TBS. The desired proteins were eluted with 1% triethanolamine, and three fractions of 5 ml each were collected. The His-tagged proteins were loaded onto an immobilized nickel nitrilotriacetic acid (Ni-NTA) column (Qiagen, Netherlands), as reported earlier (57). The purity of the proteins was assessed by sodium dodecyl sulfate-polyacrylamide gel electrophoresis (SDS-PAGE) and then dialyzed against TBS with 5 mM CaCl_2_ overnight at 4°C (purified enzymes are presented in Fig. S1 in the supplemental material). Protein concentration was estimated by absorbance (280 nm) based on the known amino acid composition of the protein using the PROTPARAM tool (58). Proteins were stored in 50% glycerol (vol/vol) at −20°C.

### Enzymatic activity assay. (i) Enzyme substrates.

Avicel, locust bean gum (for mannanase activity assay), and xylan from beech wood were purchased from Sigma-Aldrich (Darmstadt, Germany). Xyloglucan and lichenan were from Megazyme (Bray, Ireland). CMC was from Fisher Chemical (Thermo Fisher Scientific, Waltham, MA, USA). Phosphoric acid swollen cellulose (PASC) was prepared from Avicel in a manner similar to the method of Wood (59). From these substrates, fresh stock solutions were prepared with the following substrate concentration: 10% Avicel, 2% CMC, 0.6% PASC, 2% mannan, 0.5% lichenan, 2% xylan, and 1% xyloglucan.

### (ii) Activity assay.

All activity assays were performed under optimized reaction conditions (as described above): 0.5 M acetate buffer (pH 5) at 40°C. The final concentration of enzyme was 0.5 μM per reaction mixture in a total volume of 200 μl, and the final buffer concentration was 50 mM. All examined proteins were tested for their activity on all seven substrates, in different final concentrations and different incubation times: CMC, xylan, and mannan at 1% final concentration for 2 h, Avicel at 5% for 24 h, PASC at 0.3% for 24 h, lichenan at 0.25% for 1 h, and xyloglucan at 0.5% for 1 h. All assays were performed at least twice in triplicate with an agitation rate of 300 rpm. The quantification of the reduced soluble sugars released from the polysaccharide substrates was done by using the 3,5-dinitrosalicylic acid (DNS) method (60, 61). At the end of the assay, the reaction tubes were transferred to an ice-water bath and then centrifuged for 4 min at 13,000 rpm at room temperature (RT). Then, 100 μl of each sample was added to 150 μl of the DNS solution. This mixture was then incubated for 10 min at 100°C, 200 μl of this mixture was transferred to each well on a 96-well plate, and the absorbance at 540 nm was measured. The sugar concentration was determined using a glucose standard curve.

### Affinity-based ELISA of recombinant proteins.

In order to determine the cohesin-dockerin specificity of interaction, the standard affinity-based ELISA procedure was used as described previously (26). Briefly, 5 μg/ml of each dockerin-containing enzyme was immobilized on a MaxiSorp 96-well ELISA plate (Greiner Bio-One, Belgium). Subsequently, CBM-Coh modules were applied on the plate in a concentration gradient of 0.01 ng/ml to 100 μg/ml. Rabbit anti-CBD diluted 1:5,000 was used as the primary antibody preparation. The negative control was Cel5D-*Ct* (a dockerin-containing enzyme from *C. thermocellum*) (62, 63). The pEC_50_ values were calculated using the “Quest Graph EC50 calculator” (https://www.aatbio.com/tools/ec50-calculator/) of AAT Bioquest, Inc. (Sunnyvale, CA, USA).

### Affinity electrophoresis.

The affinity electrophoresis assay was performed by the method of Moraïs et al. (28). In this assay, we tested six different gels that contained various polysaccharides in different percentages: (i) 0.1% (wt/vol) PASC, (ii) 0.1% (wt/vol) xyloglucan, (iii) 0.2% (wt/vol) xyloglucan, (iv) 0.2% (wt/vol) xylan, (v) 0.2% (wt/vol) starch, and (vi) 0.1% (wt/vol) mannan. Each gel was run for 2.5 h at 100 V, versus a control gel (with no polysaccharide). Relative mobilities were calculated (and normalized) versus BSA mobility on the control gel.

### Affinity pulldown.

The affinity pulldown assay is based on measuring the soluble protein concentrations in a sample containing an insoluble polysaccharide versus a reference sample without the polysaccharide. Each sample was prepared in a 1.5-ml tube and contained 25 mg Avicel, a protein with a final concentration of 0.1 mg/ml, 100 μl TBS×10, and double distilled water up to 1 ml. A reference sample was prepared the same but without Avicel. All samples were shaken on rotary rotator at 4°C for 1 h. The samples were then centrifuged at 13,000 rpm for 5 min, and the supernatant fluids (“unbound” fraction) were separated from the pellet (“bound” fraction). Each pellet was washed three times using TBS×1 buffer (pH 7.4), and resuspended with 225 μl TBS×1. The protein concentration was measured from the supernatant, pellet, and reference fractions, using the Pierce BCA protein assay kit (Thermo Scientific, USA). The positive control was CBM3 of *C. thermocellum* (27). All the measurements were performed at least three times. Reduction in protein concentration compared to the reference sample indicates binding to Avicel.

### Anaerobic fermentation of Clostridium saccharoperbutylacetonicum.

*C. saccharoperbutylacetonicum* strain N1-4 (HMT) (DSM 14923) was obtained from the German Collection of Microorganisms (DSMZ, Leibniz, Germany). The microorganism was grown on a tryptone-yeast extract-acetate (TYA) medium under anaerobic conditions by the method of Al-Shorgani et al. (64) with either 4% (wt/vol) cellobiose (CB) (Sigma Chemical Co., St. Louis, MO) or 5% (wt/vol) brewer’s spent grain (BSG), kindly provided by Instituto Tecnológico Agrario de Castilla y León (ITACyL) Valladolid, Spain (34), as the sole carbon and energy source. Growth on each of the two carbon sources was performed in two biological repeats. *C. saccharoperbutylacetonicum* cell culture supernatant fluids were filtered through 0.2-μm sterile plastic filters (Thermo Fisher Scientific, Waltham, MA, USA) and concentrated 15-fold using a Vivaspin concentrator (30-kDa cutoff; Sartorius Stedim Biotech GmbH, Göttingen, Germany).

### Affinity-based ELISA for detection of *in vivo* cellulosome.

Affinity-based ELISAs were performed as described above with minor modifications. For affinity-based ELISA of material absorbed to plates, 96-well ELISA plates were coated with concentrated spent cell culture media from CB- or BSG-grown *C. saccharoperbutylacetonicum* cells or with recombinant scaffoldin (used as the positive control). Next, 10 μg/ml XynDoc44 was used to examine the presence of the scaffoldin (via cohesin-dockerin interaction). Rabbit anti-XynT6 diluted 1:5,000 was then used as the primary antibody, followed by HRP-labeled anti-rabbit diluted 1:10,000 as the secondary antibody (26).

For the affinity pulldown assay, the concentrated spent cell culture media from CB- and BSG-grown *C. saccharoperbutylacetonicum* were incubated with 25 mg of Avicel. Here, the washed incubated “Avicel pellet” was interacted with XynDoc44 followed by the above-described immunochemical procedure. Negative-control experiments were performed without the XynDoc44 interaction step. All experiments were performed at least twice.
