# Supplementary figures and images for: Minimalistic Cellulosome of the Butanologenic Bacterium Clostridium saccharoperbutylacetonicum
Source: mBio. 2020 Mar 31;11(2):e00443-20. doi: 10.1128/mBio.00443-20 (PMC7157769; doi:10.1128/mBio.00443-20)

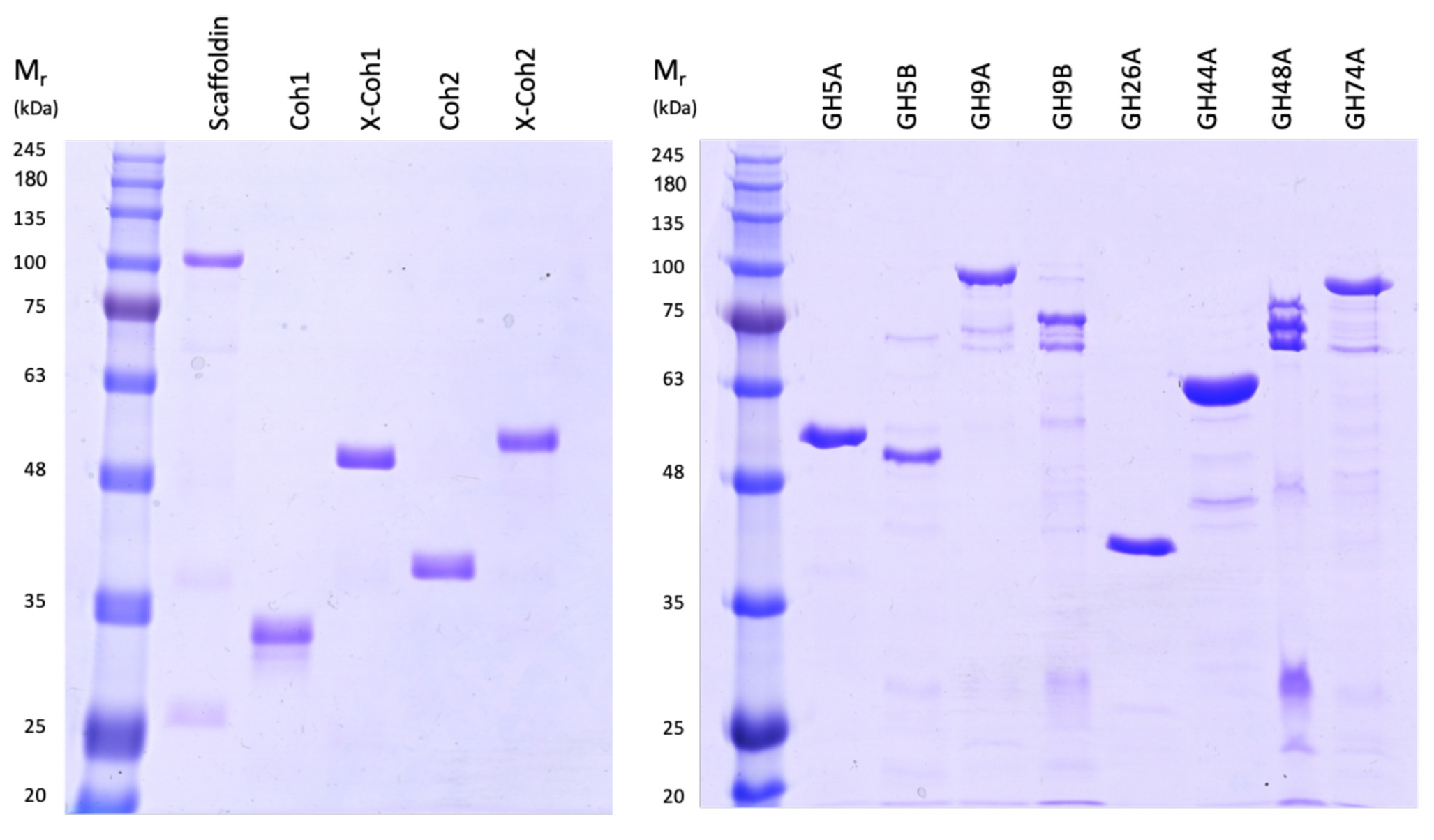

Supplement: FIG S1 [file mBio.00443-20-sf001.jpg]

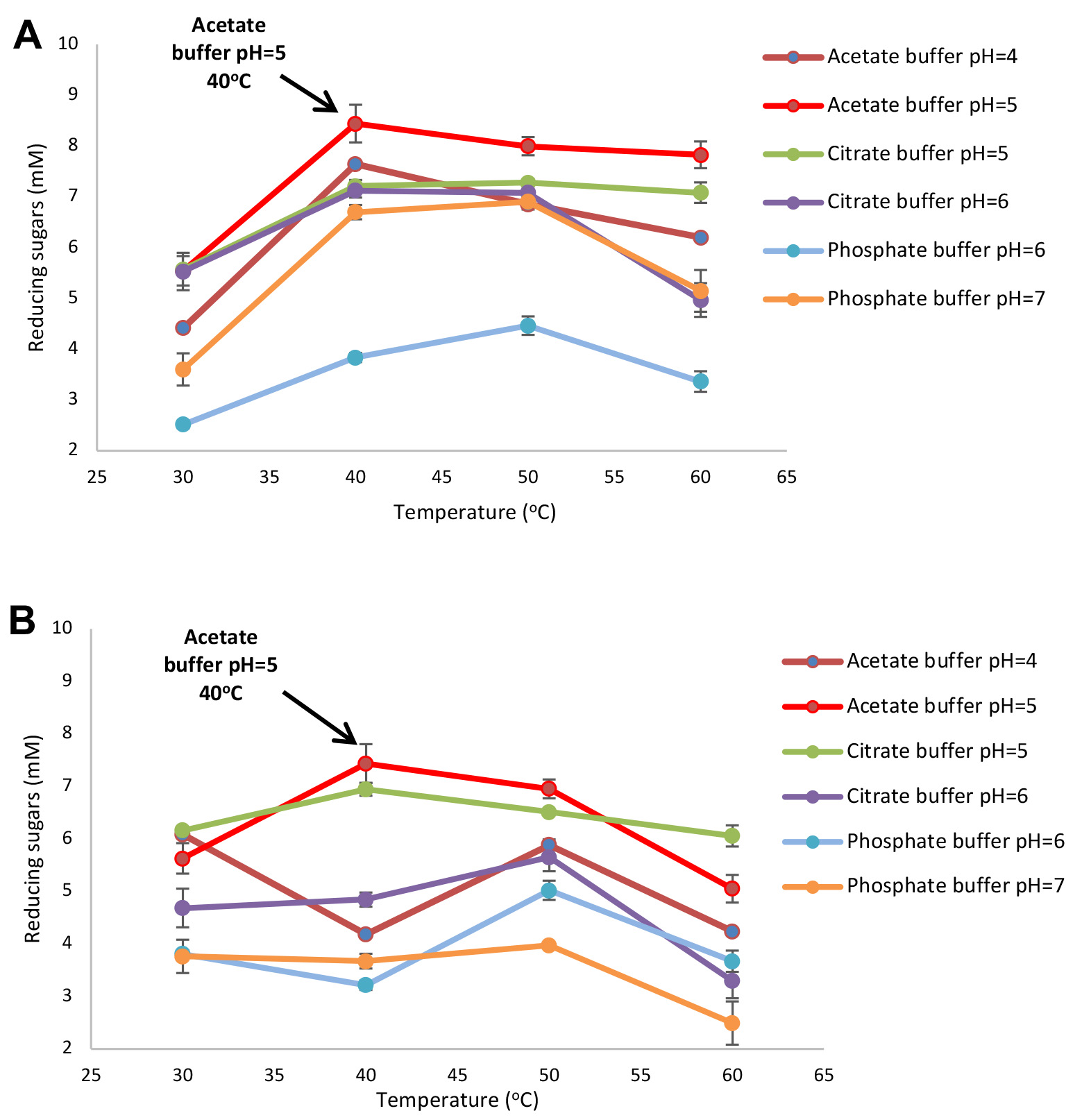

Supplement: FIG S2 [file mBio.00443-20-sf002.jpg]

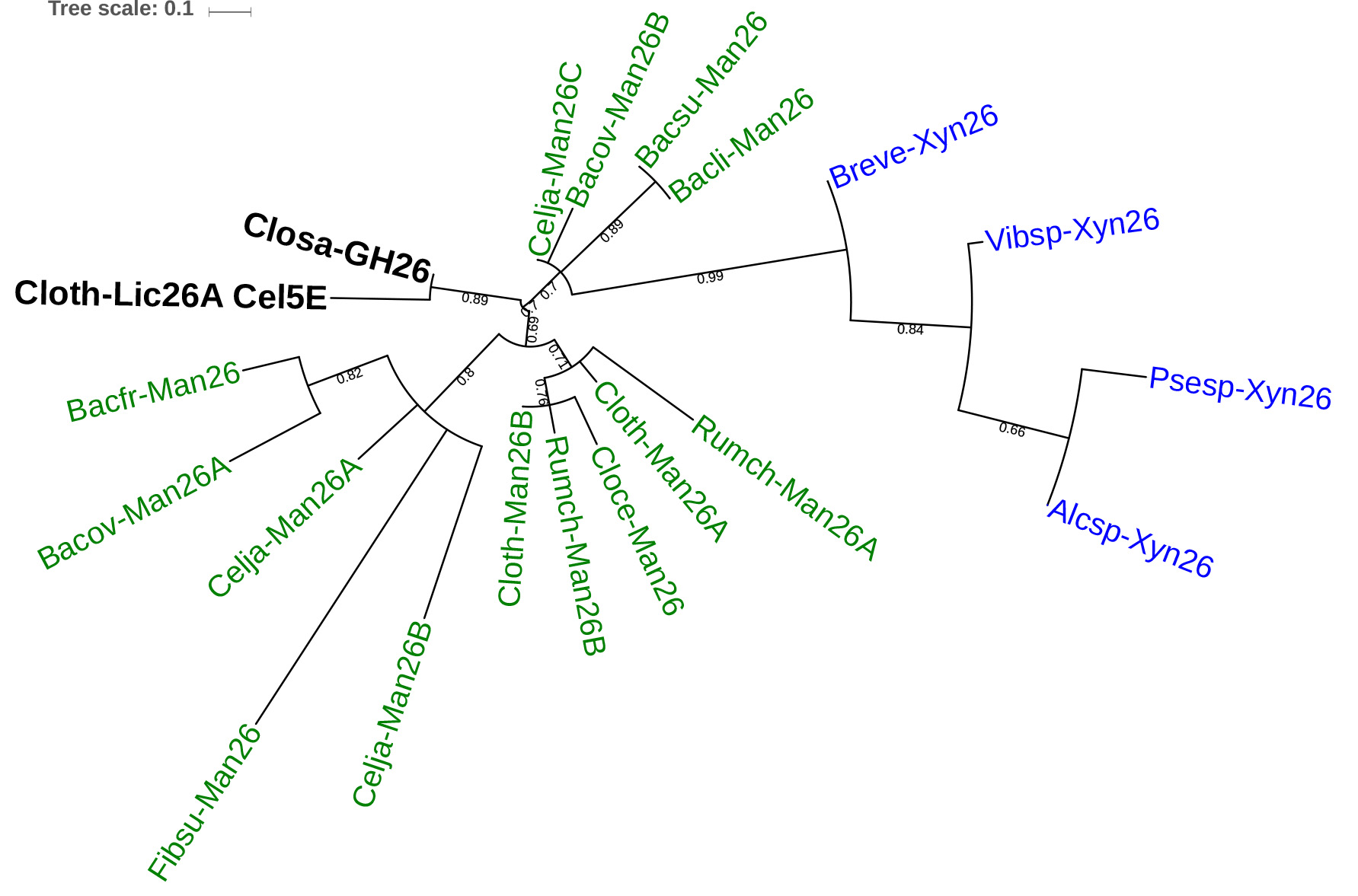

Supplement: FIG S3 [file mBio.00443-20-sf003.jpg]

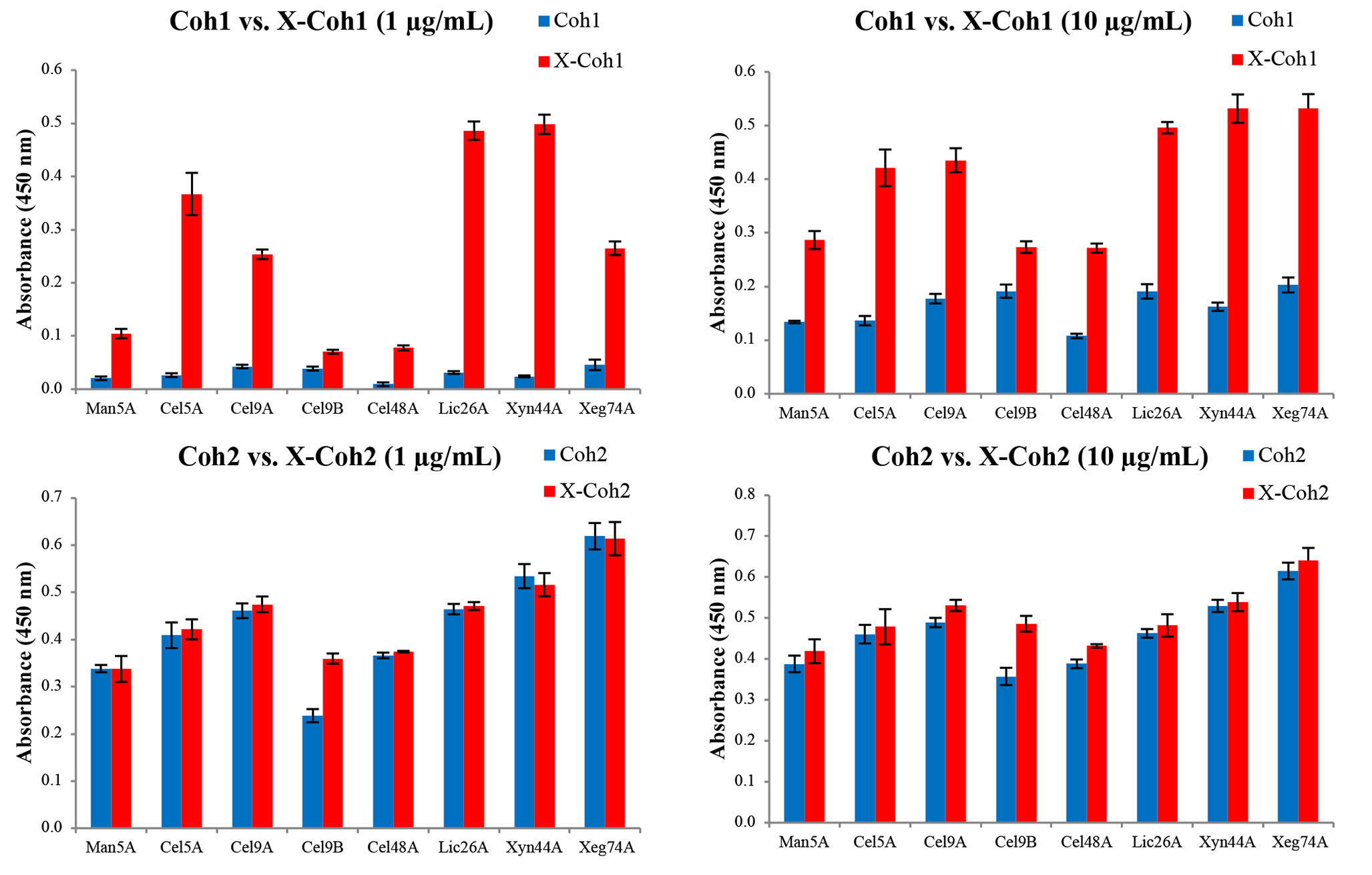

Supplement: FIG S4 [file mBio.00443-20-sf004.jpg]

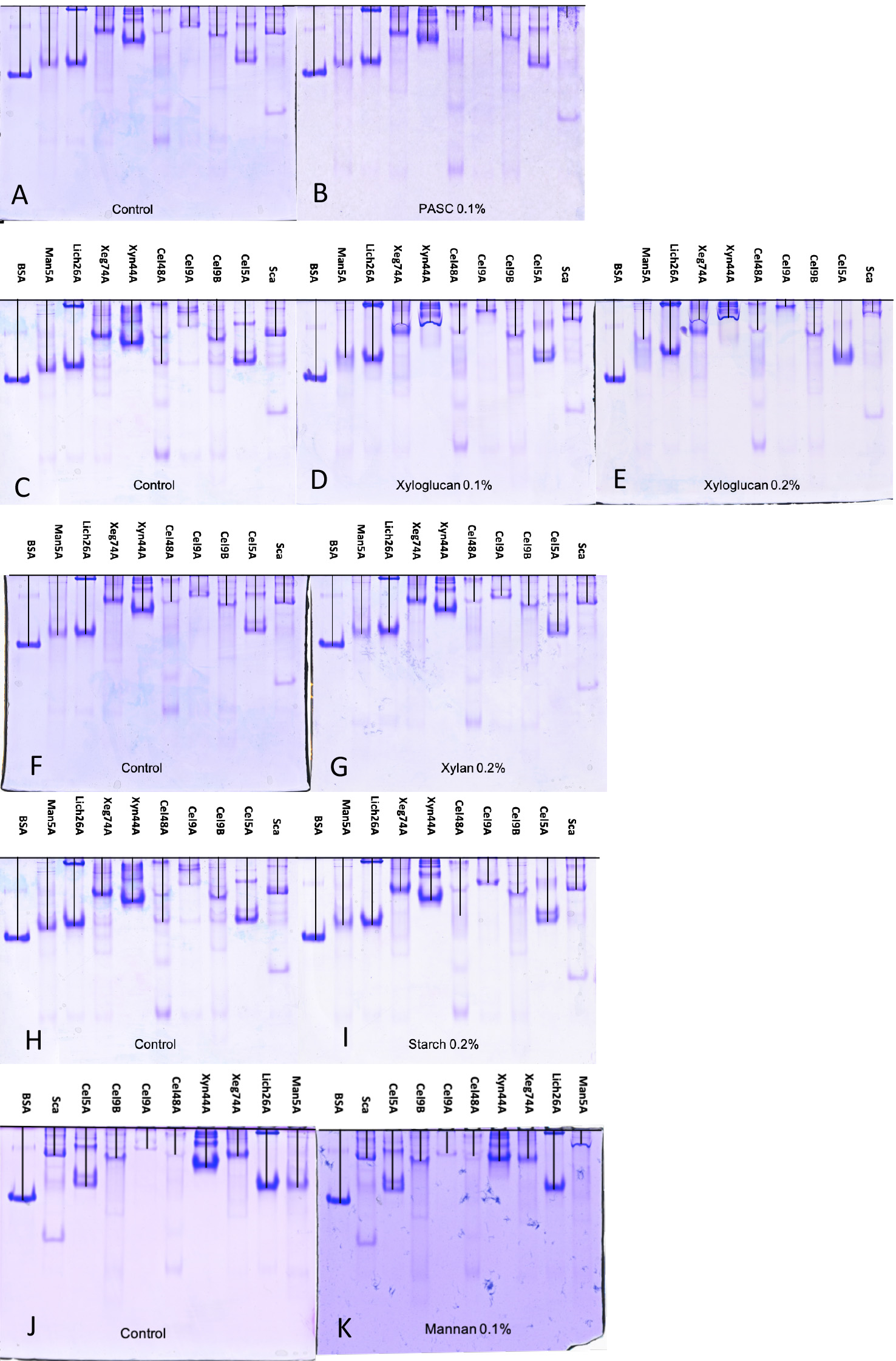

Supplement: FIG S5 [file mBio.00443-20-sf005.jpg]

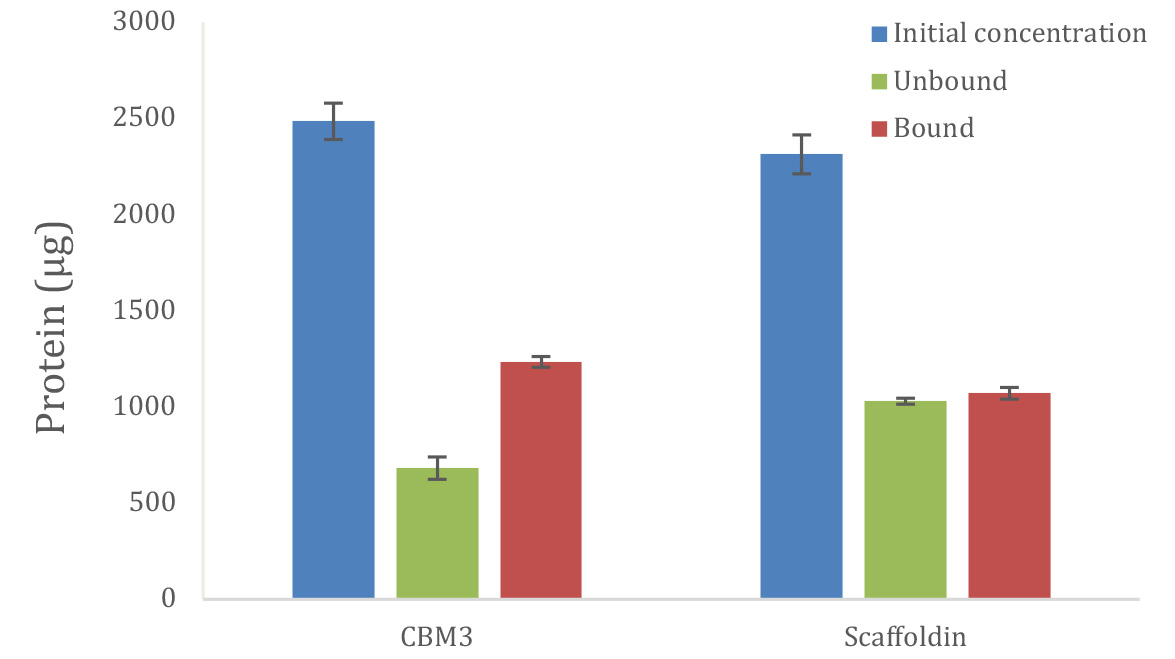

Supplement: FIG S6 [file mBio.00443-20-sf006.jpg]

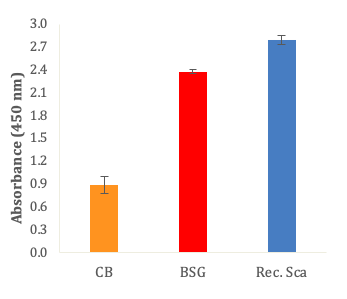

Supplement: FIG S7 [file mBio.00443-20-sf007.tif]
