# Supplementary material for: Minimalistic Cellulosome of the Butanologenic Bacterium Clostridium saccharoperbutylacetonicum
Source: mBio. 2020 Mar 31;11(2):e00443-20. doi: 10.1128/mBio.00443-20 (PMC7157769; doi:10.1128/mBio.00443-20)
Supplement: TABLE S1 [file mBio.00443-20-st001.pdf]

Table S1: Primers used in the study (restrictions sites represented in upper cases)

Primer	Sequence
F-GH5A-NcoI	atattaCCATGGgcgcagaaaatgataatagtgatg
R-GH5A-XhoI	tatattCTCGAGttgtaaaattaatttttttaaac
F-GH5B-RF*	AATTTTGTTTAACTTTAAGAAGGAGATATACCATGGgcgcagaaataagtccgattaattcaagta
R-GH5B-RF*	AGCCGGATCTCAGTGGTGGTGGTGGTGGTGCTCGAGtccctctaaaattatttttcttaatgcaaa
F-GH9A-RF*	AATTTTGTTTAACTTTAAGAAGGAGATATACCATGGgcgcagaaataagtccgattaattcaagta
R-GH9A-RF*	AGCCGGATCTCAGTGGTGGTGGTGGTGGTGCTCGAGactatttataattgttttccttaatgcaaa
F-GH9B-NcoI	atattaCCATGGgcgatgaaagcagcataaaatac
R-GH9B-XhoI	tatattCTCGAGttgttcaattatggttcttct
F-GH26A-NcoI	atattaCCATGGcgagtactgagattcaaaacaatag
R-GH26A-XhoI	tatattCTCGAGttctaaaattaatttttttcaaaac
F-GH44A-NcoI	atattaCCATGGgcgaagagacaaattctattaacataag
R-GH44A-XhoI	tatattCTCGAGttcatttatcttttttcttagttc
F-XynT6-GH44Doc-KpnI	tattaggtaccgCAAGGTTCAACAACTATGCC
R-XynT6-GH44Doc-BamHI	tactatggatccTTATTCATTTATCTTTTTTCTTAGTTC
F-GH48A-NcoI	atattaCCATGGgcgctactacagctcaaaatgcttcg
R-GH48A-XhoI	tatattCTCGAGttgattatccaataccatttttct
R-GH74A-XhoI	tatattCTCGAGactattaattatttttcttaaagc
F-GH74A-NcoI	atattaCCATGGcggaaacaacaaacaatgttgaac
F-put-Sca-NcoI	atattaCCATGGgcatgaaaagaaaaaaaaattttaagcg
R-put-Sca-XhoI	tatattCTCGAGtttaacctctatacttccattattaaaatc
F-CBM-Coh1-BamHI	atattaGGATCCgcagttgaagatggtttagtt
R-CBM-Coh1-XhoI	tatattCTCGAGtcatccaatagttatttttccaacc
F-CBM-Coh2-BamHI	atattaGGATCCgctactgaaacattggctgcaggc
R-CBM-Coh2-XhoI	tatattCTCGAGttatttaacctctatacttcc
F-CBM-x-Coh1-BamHI	atattaGGATCCactgactctaatgtaacgccagatg
R-CBM-x-Coh1-XhoI	tatattCTCGAGtcatccaatagttatttttccaacc
F-CBM-x-Coh2-BamHI	atattaGGATCCggagatggtggtgtagtaacacctg
R-CBM-x-Coh2-XhoI	tatattCTCGAGttatttaacctctatacttcc
**Internal primers for DNA-sequencing**	
Int1-GH9A	CCAGGATACGATAAAGAACC
Int2-GH9A	GGTAGATCTTAAGAGAGCTCC
Int-GH9B	GATGAGCTTTCATGGGCTGC
Int-GH44A	GGTAAATCAGATACTGCAACAGG
Int1-GH48A	GGAAAACAGTACCTCAACCATCC
Int1-GH74A	TGCAACTTGGTCAGCTGTTG
Int2-GH74A	GAAGAGGAAGCTGTAAGTGC
Int1-Sca	GGGTTCAGCAGTTGCTAATGC
Int2-Sca	GAGTCAAGGTACAGACTATG

*RF- restriction free
